# The Tri-State Dental Meeting

**Published:** 1894-03

**Authors:** 


### The Tri-State Dental Meeting.

At the last annual meeting of the Ohio State Dental Society a proposition was offered that in 1895, at some time to be agreed upon, the States of Indiana, Michigan and Ohio hold their annual meeting in joint session, at some place to be hereafter decided upon. The proposal met with general favor, and a committee was appointed to confer with the officers or a special committee of the State Societies of Indiana and Michigan with the view of making arrangements for such a meeting, especially if the proposition should be entertained as favorably by these two Societies as it was by Ohio.

Such a meeting would be of great interest and profit, would be one which, if properly arranged for, should bring together the leading members of the profession of the three States.

This simple mention is made of this matter in order that it may be in the minds of those interested. We hope to be able to speak more definitely about it within the next few months.

